# Results of a phase I/II clinical trial: standardized, non-xenogenic, cultivated limbal stem cell transplantation

**DOI:** 10.1186/1479-5876-12-58

**Published:** 2014-03-03

**Authors:** Nadia Zakaria, Tine Possemiers, Sorcha Ní Dhubhghaill, Inge Leysen, Jos Rozema, Carina Koppen, Jean-Pierre Timmermans, Zwi Berneman, Marie-Jose Tassignon

**Affiliations:** 1 Centre for Cell Therapy and Regenerative Medicine, Antwerp University Hospital, Edegem 2650, Belgium; 2 Dept of Ophthalmology, Antwerp University Hospital, Edegem 2650, Belgium; 3 Department of Veterinary Sciences, University of Antwerp, Edegem 2650, Belgium

**Keywords:** Limbal stem cell transplantation, Clinical trial, Amniotic membrane, Tissue specific stem cells, Tissue regeneration, Cell transplantation, Cellular therapy, Cell culture, Progenitor cells, Somatic stem cells, Limbal epithelial stem cells, Corneal reconstruction, Ocular surface reconstruction, Corneal neovascularization, Corneal opacity, SHEM, CNT-20, Composite grafts

## Abstract

**Background:**

To determine if a standardized, non-xenogenic, reduced manipulation cultivation and surgical transplantation of limbal stem cell grafts is a safe and effective treatment option for patients with total and partial limbal stem cell deficiency.

**Methods:**

In vitro cellular outgrowth and phenotype of the limbal epithelial cell and composite grafts were validated using a new protocol. Patients received either autologous (n = 15) or allogenic (n = 3) explants cultured using a standardized protocol free from xenogenic products. The resulting grafts were transplanted using a reduced manipulation surgical technique.

**Results:**

The majority of cells (>50%) displayed a progenitor phenotype typified by positive immunofluorescence for ∆Np63, CK14 and ABCG2 and low immunofluorescence for CK3/12 and desmoglein 3 proteins. The surgical protocol was designed to minimize manipulation and the graft itself was secured without sutures. The transplant recipients were followed for a mean of 24 months. Twelve of the 18 transplant recipients were graded as anatomically successful (67%), based on the defined success parameters. There was a significant reduction in corneal neovascularization, which was accompanied by an improvement in pain though not photophobia or central corneal opacity post transplant. The transplantation protocol showed no measureable effect on visual acuity.

**Conclusion:**

We conclude that this standardized culture system and surgical approach is safe and effective in reducing corneal neovascularization. The technique is free from animal contaminants and maintains a large proportion of progenitor cells. Although this technique did not improve visual function, restoring a functional epithelial cell layer and reducing corneal neovascularization provides an improved platform for a penetrating keratoplasty to ultimately improve visual function.

## Introduction

Limbal stem cell deficiency (LSCD) can result from a range of pathologies including ocular cicatricial pemphigoid, Stevens Johnson syndrome, aniridia, multiple surgeries and trauma [1]. The limbus is depleted of the resident epithelial stem cells permitting a vascular conjunctival membrane to grow over the cornea resulting in scarring, poor vision, pain and photophobia. These patients are a high-risk group for treatment with vision restoring therapies such as penetrating keratoplasty (PK) [2]. Limbal stem cell deficiency is an orphan pathology, which prior to 1998 had limited therapeutic options. Transplantation may be performed either by directly implanting a kerato-limbal graft or by harvesting a biopsy, expanding the cells by tissue culture and then transplanting the graft [3]. The advantage of the latter is that it requires a smaller volume of donor tissue, reducing the risk of LSCD in the donor eye [4]. The emergence of clinical trial data supporting the benefit of limbal stem cell transplantation has led to its more widespread use [5-14], and as transplantation procedures increase, the need for optimization and standardization of the technique comes to the fore. In this paper we present the results of a clinical trial that contains data on the additional steps we have taken in seeking a more ideal transplantation protocol.

Our primary aims were to standardize the limbal stem cell protocol where possible, remove any animal derived product and to apply a minimal manipulation surgical protocol in order to improve clinical outcomes. An alternative medium was assessed, a means of amnion membrane fixation used and the surgical procedure optimized to simplify transplantation and reduce manipulation and suture influence. Post-operative outcome assessments included quantitative measurements of corneal neovascularization, opacity and visual acuity and subjective reports of pain and photophobia. Clinical examination was performed to assess graft integrity and anatomy. All of these factors were assessed to determine the safety and efficacy of the protocol.

## Materials and methods

The study was approved by the Antwerp University Hospital Ethical Committee (approval number: EC7/28/153; EudraCT no 2008-001543-19) and followed the tenets of the Declaration of Helsinki. Written informed consent was obtained from all participants after explanation of the procedure and possible side effects. Patients that were pregnant or lactating, suffering from severe psychological disorders, or had active inflammation of the eye were excluded from the study.

### Animal-product free culture protocol validation

Progenitor cell targeted (PCT) CNT-20 media (CellnTec, Switzerland) supplemented with 1% human type AB serum was compared with supplemental hormonal epithelial medium (SHEM) consisting of DMEM/F12 supplemented with 5% fetal bovine serum (FBS), 50 μg/ml gentamycin, 1.25 μg/ml amphotericin B, 5 μg/ml insulin-transferrin-selenium growth supplement (all from Gibco, Invitrogen, Belgium), 5% DMSO, 30 ng/ml cholera toxin, 0.5 μg/ml hydrocortisone (all from Sigma Aldrich, Diegem, Belgium) and 2 ng/ml epidermal growth factor (Millipore, MA, USA).

Human corneo-scleral tissue (n = 5) was obtained from the cornea tissue bank (UZA, Belgium) and two limbal explants were obtained from each eye (total n = 10), Limbal stem cell amnion grafts were generated using methods described previously [15]. The membranes were prepared on interlockable plastic rings as described below. Briefly, the explants were cultured for 14 days at the air liquid interface in either CNT-20 (n = 5) or SHEM (n = 5) after which they were fixed in 4% paraformaldehyde for one hour. Tissue whole mounts were prepared and stained with primary antibodies, used in accordance with the manufacturers’ instructions, against collagen IV (Abcam: ab6586, dilution 1/100), integrin α6 (Abcam: ab20142, 1/50), collagen II (Abcam: ab34712, 1/100), laminin (Abcam: ab11575, 1/25), connexin 43 (Millipore: MAB3068, 1/50), ∆Np63 (Santa Cruz Biotechnology: sc-8609, 1/300). All samples were observed and photographed using a laser scanning confocal microscope (LSM510, Carl Zeiss).

**Figure 1:**
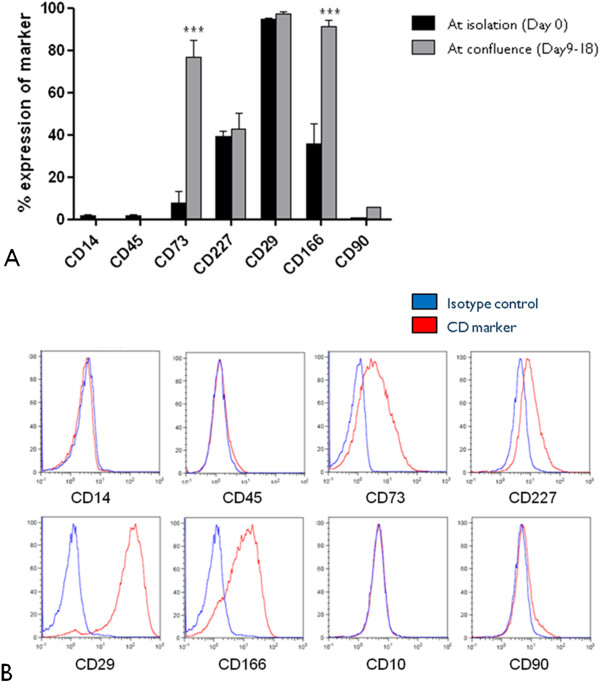
**Flow cytometry profiles of limbal epithelial cells grown in CNT-20 medium.** LEC cytometry profiles at isolation and at confluence **(A)**, phenotypic profile of LECs at confluence **(B)** (***p < 0.001).

Freshly isolated limbal epithelial cell (LEC) phenotypic profiles were compared to that of LECs grown to confluence in CNT-20 media with respect to expression of hematopoietic markers (CD14, CD45), markers of stemness (CD73), secretory markers (CD227/MUC1), fibroblast markers (CD10, CD90) and adhesion molecules (CD29/integtin β1, CD166/ALCAM) using flow cytometry (Figure 1). LEC profiles were also compared with epithelial cells harvested from cadaveric corneas. Results were analyzed in FlowJo version 8.8.4 for Macintosh and statistical analysis performed using GraphPad Prism 5.

### Diagnosis and classification of limbal stem cell deficiency

The diagnosis of LSCD was made clinically. The presence of conjunctivalizaton (one or more signs: corneal vascularization, corneal epithelial defects, scarring, late fluorescein staining) with the absence of normal limbal architecture (limbal palisades of Vogt) was indicative of LSCD. Ocular surface photographs were taken to document the corneal neovascularization, opacity and epithelial defects. Impression cytology was performed as an adjunct to confirm the diagnosis (data not shown) [16]. Visual acuity was recorded using the ETDRS chart and where patients had visual impairments of counting fingers, hand movements a numerical conversion was performed as described [17]. Light perception was registered numerically as a visual acuity of 0. Pain was recorded using a numerical pain scale (0–10, 0 corresponding to no pain and 10 corresponding to the worst pain possible in the verbal descriptor scale) [18]. Photophobia was recorded similarly using a 4 point scale (0 = nil photophobia, 1 = mild, 2 = moderate and 3 = severe photophobia). Schirmer’s test 1 was performed to ensure the presence of an adequate tear film. Patient with values <5 mm in 5 minutes required punctal plugs prior to surgery. The complete absence (360˚) of limbal architecture with conjunctivalization was termed total limbal stem cell deficiency. The involvement of fewer clock hours was considered to be partial deficiency.

### Limbal biopsy

The limbal biopsy was harvested under local anesthesia from the contralateral eye in cases of unilateral disease (n = 15) and from a HLA (>50%) matched living related donor in bilateral disease (n = 2) and one unmatched cadaveric donor (n = 1). For the living related donors, a three loci match of at least 50% was favored taking into account HLA-A, HLA-B and HLA-DR. A superficial area of 1 mm by 2 mm was harvested from either the superior or inferior limbus using a 45˚ diamond blade to delineate the biopsy and a crescent knife to tunnel to a depth of approximately 100 microns. The biopsy was placed in PCT CnT-20 medium and transported to the laboratory where it was rinsed with culture medium containing antibiotics.

### Standardized generation of limbo-amnion composite graft

The human amnion membrane (HAM) was standardized by removal of a thick, hydrophilic, spongy layer and amniotic epithelial cells as previously reported [15,19,20]. The resulting membrane was secured with two interlockable plastic amnion rings (35 mm Lumox cultureware, Greiner Bio-One, BeLux) with the basement membrane facing upwards. The interlockable ring design ensures that the HAM remains oriented and smooth from culture to transplantation. The limbal biopsy was placed on the membrane and cultured in corneal progenitor cell targeted CnT-20 medium supplemented with 1% human blood type AB serum (Sigma Aldrich, Germany) at the air liquid interface at 37˚C and 5% CO_2_. Limbal explants were cultured for 14 days under these conditions with the medium changed every 2 to 3 days. The culture was monitored for cell morphology and the diameter of the cell outgrowth recorded. An outgrowth of >8 mm in diameter with limbal epithelial cell morphology and negative running test results for microbes (aerobes, anaerobes, fungi and mycobacterium) allowed release of the composite graft for transplantation.

### Immunofluorescent labeling of composite grafts

Cultured graft whole mounts were labeled with anti-CK14 (Chemicon: CBL197; dilution 1/50), anti-BCRP/ABCG2 (Calbiochem: OP191; 1/50) and anti-p63 antibody (Thermo Scientific: rb-9424-R7;1:1) to determine the presence of corneal epithelial progenitor cells. Anti-desmoglein 3 (Abcam, AB14446; 1/200) and anti-CK 3/12 (Chemicon:CBL218; 1/50) were used to establish the presence of terminally differentiated corneal epithelial cells. The presence of vascular endothelial growth factor receptors VEGF-R1 (Epitomics: 1303–1, 1/250) and VEGF R3/FLT (Santa Cruz: Sc-28297, 1/50) was also investigated (data not shown). Normal human corneal cross sections were routinely used as negative controls where the primary antibodies were omitted from the staining protocol. The samples were observed and photographed under a Laser Scanning Confocal Microscope LSM510 Duphoton (Carl Zeiss).

### Surgical technique: cultivated limbal stem cell transplantation

The compostie grafts were transplanted using a minimal manipulation technique. [15] Briefly, a 360˚ conjunctival periotomy was performed followed by dissection of the fibrovascular pannus. In cases of partial limbal stem cell deficiency, only the localized pannus was dissected beyond the limbus. Fibrin glue (TissueCol®) was applied to the surface of the cornea and the graft, secured within the plastic rings, was positioned. This was done by grasping the plastic rings and lowering the construct onto the denuded cornea, without touching the graft itself. Once the glue had set, the graft was cut out of the rings just beyond the limbs. A secondary HAM was placed over the graft (spongy layer facing up), tucked under the free edges of the conjunctiva and secured using four 10–0 nylon sutures. This membrane served as a temporary membrane patch. A bandage contact lens was applied and left in place until the removal of all sutures one-week post transplant.

### Post-operative medication

Autologous transplant recipients were prescribed the following regimen: 0.3% ofloxacin drops 4×/day, prednisolone drops 8×/day, autologous serum drops 8×/day continued for one month and then gradually tapered. Allogenic transplant recipients were also prescribed oral cyclosporine A 2×125 mg/day (commenced 1 week prior to transplantation maintaining blood trough levels at approx 100 to 150 ng/ml), solu medrol 125 mg IV peri operatively, continued for 3 days post op then switched to methylprednisolone 1 mg/kg/day. Systemic immunosuppression was tapered and discontinued at one-year post transplantation.

### Definition of successful outcome parameters

Success was judged based on two factors: 1. Reversion to a persistent intact epithelium was deemed an ‘anatomical success’ and 2. A functional improvement in pain, photophobia and visual acuity was termed ‘functional success’.

### Quantifying corneal neovascularization and opacity

Ocular surface photographs were analyzed using our previously published technique for quantifying corneal neovascularization [21]. The degree of corneal opacification was assessed by taking slit-lamp photographs and analyzing the images within Matlab. A circular region in the central 25% of the cornea was examined. Corneal backscatter in severe opacification is mostly in the blue-white hue and it was elected to use the average intensity of the light in the blue channel for quantifying central corneal opacity.

### Statistics

The efficacy of cultivated limbal epithelial transplantation was examined by comparing the outcome parameters (corneal neovascularization, corneal opacity, pain and photophobia) pre and post transplantation by Wilcoxon signed rank tests. The overall success percentage together with a 95% confidence interval was calculated. Success outcomes were based on the total group. Post-operative improvements were assessed for the entire group and also in the subgroup of patients in whom the grafts achieved stability and were deemed anatomical success. Visual acuity was compared pre and post transplantation with a sign test. The influence of gender and etiology on the success rate of LSCD was assessed with the Fischer Exact Test. In addition, a linear regression analysis was performed to examine the effect of age, gender and etiology on the outcome parameters. All analyses were performed using SPSS 20.2 (IBM Statistics Inc, Chicago IL, USA) at a significance level of 0.05.

## Results

### Animal product-free culture validation

**Figure 2:**
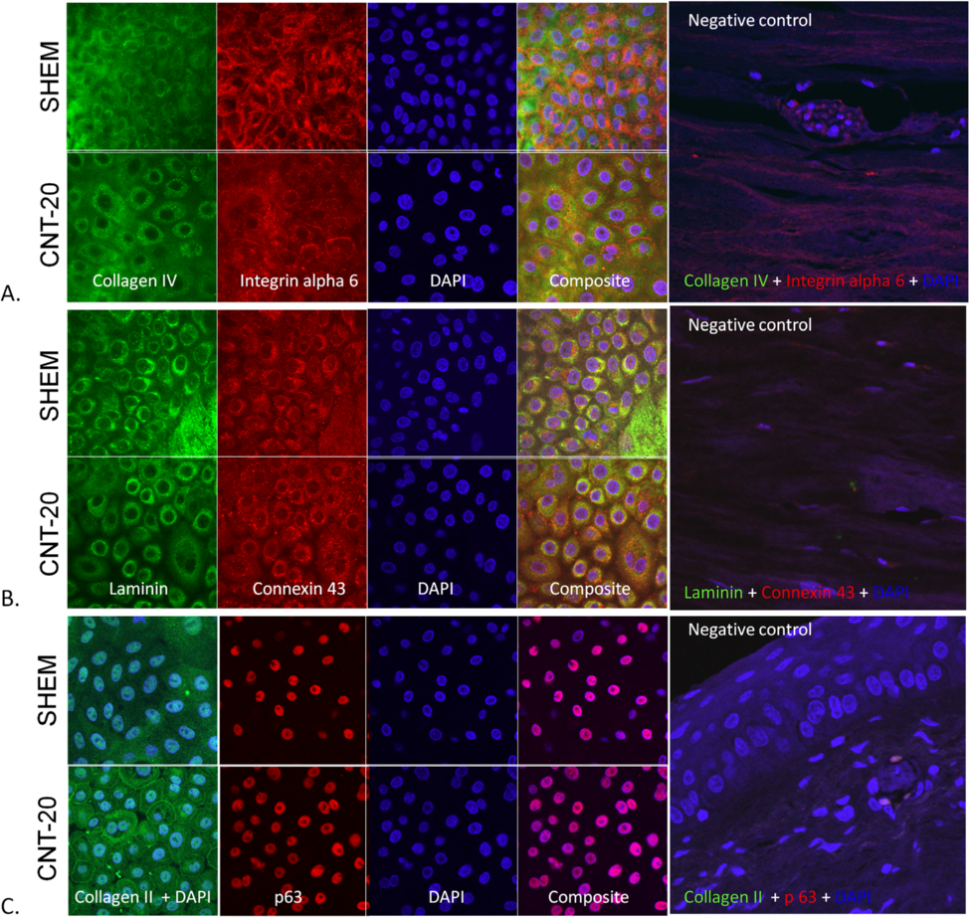
**Limbal epithelial cells cultivated on amniotic membrane in progenitor cell CNT-20 versus SHEM medium.** LECs grown in CNT20 and SHEM stained for the expression of Collagen IV and Integin alpha 6 **(A)**, Laminin and Connexin 43 **(B)** and Collagen II and ∆Np63 **(C)**.

The initial media validation experiments confirmed cellularization of the HAM with cobblestone-like appearance of LECs in both SHEM and progenitor cell targeted CNT-20 medium. Both CNT-20 and SHEM showed a similar expression for ∆Np63 and extracellular matrix proteins: collagen IV, laminin and connexin 43 (Figure 2). The expression of integrin α6 was positive though reduced in the CNT-20 cultures when compared with SHEM cultures. In the CNT-20 cultures, collagen II was highly expressed in the cell membranes with weak cytoplasmic expression, but minimal expression was observed in the SHEM cultures. LECs cultivated in progenitor cell targeted CNT-20 medium were negative for the hematopoietic markers: CD14, CD45 and fibroblast markers: CD10, CD90 while positive for stemness: CD73, secretory CD227, adhesion molecules: CD29, CD166. Moreover, cells cultivated to confluence in CNT-20 displayed a significantly higher expression of stem cell marker CD73 (p > 0.001) and adhesion molecule CD166 (p > 0.001) when compared with freshly isolated epithelial cells from donor cadaveric corneas (Figure 1). Initial experiments displayed adequate expansion of LECs in CNT-20 serum-free medium on plastic cultureware but this could not be replicated with LECs on HAM (data not shown).

### Pre-operative assessment and recruitment

**Figure 3:**
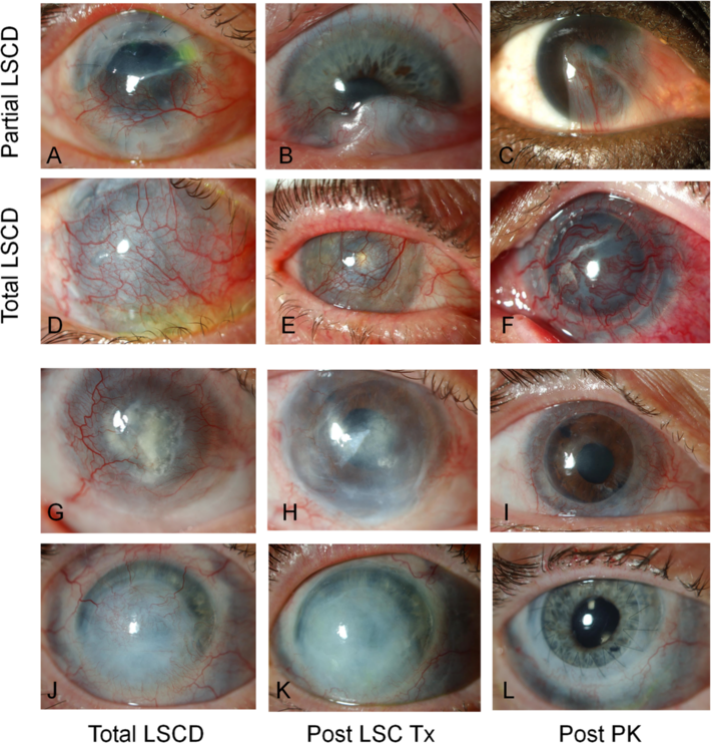
**Preoperative images.** All three partial deficiency patients are shown **(A-C)** and three representative total deficiency patients **(D-F)**. Representative images of two patients with total limbal stem cell deficiency prior to **(G, J)** and following **(H, K)** limbal stem cell transplantation. The same eyes following penetrating karatoplasty **(I, L)**.

Eighteen patients with LSCD were enrolled in the clinical trial, 11 males and 7 females with a mean age of 40.6 years (range 6–79). Sixteen patients had unilateral and 2 had bilateral disease. Out of the 18 patients enrolled, 15 were diagnosed with total and 3 with partial limbal stem cell deficiency. The three partial limbal stem cell deficient participants are shown (Figure 3A-C) with representative images of the total LSCD patients (Figure 3D-F). The most common cause for the limbal stem cell deficiency was chemical burn (n = 7). Other causes are listed in Table 1.

**Table 1:** Overview of patients enrolled within the clinical trial

**Total LSCD**									
**No.**	**AGE**	**SEX**	**LSCD etiology**	**Type**	**Follow-up (months)**	**VA before**	**VA after**	**Tx Post LEC TX**	**Result**
**1**	44	M	Chemical burn	allo* auto	41	CF (0.014)	CF (0.014)	PK	Failed
**2**	45	F	Chemical burn	auto	43	HM (0.005)	0,05	-	AS, FS
**4**	45	M	Chemical burn	auto	31	CF (0.014)	<0,05	-	Failed
**5**	6	M	Chemical burn	auto	31	HM (0.005)	LP (0)	PK	Failed
**6**	27	M	Aniridia	allo**	27	0.053	0.1	PK	AS, FS
**7**	70	F	Corneal ulcer	auto	25	LP (0)	HM (0.005)	PK	AS, FS
**8**	29	M	Chemical burn	auto	25	<0,05	HM (0.005)	-	Failed
**10**	33	M	Chemical burn	auto	19	LP (0)	LP (0)	-	Failed
**11**	63	M	Iatrogenic	auto	19	HM (0.005)	HM (0.005)	-	AS
**13**	31	F	Aniridia	allo**	48	CF (0.014)	0.2	PK	AS, FS
**14**	16	F	Kniest syndrome	auto	6	LP (0)	HM (0.005)	-	AS
**15**	6	M	Keratitis	auto	5	LP (0)	LP (0)	-	Failed
**16**	48	M	Chemical burn	auto	4	CF (0.014)	0.125	-	AS, FS
**17**	79	M	Trauma	auto	4	CF (0.014)	CF (0.014)	-	AS
**18**	42	F	Viral keratitis	auto	18	LP (0)	HM (0.005)	PK	AS, FS
**Partial LSCD**									
**No.**	**AGE**	**SEX**	**LSCD Etiology**	**Type**	**Follow-up (months)**	**VA before**	**VA after**	**Tx Post LEC TX**	**Result**
**3**	57	F	Iatrogenic	auto	39	0.15	0,16	-	AS, FS
**9**	44	F	Keratitis, uveitis	auto	24	CF (0.014)	0.15	PK	AS, FS
**12**	47	M	Trauma	auto	18	0.2	0.9	-	AS, FS

The table shows the subject code, age, sex, etiology of LCSD (Limbal Stem Cell Deficency), type of donor (allo* = allogenic cadaveric, allo** = from living related donor), the follow-up, visual acuities pre and post op (LP = light perception, CF = counting fingers, HM = hand movements) surgical interventions following LEC TX (Limbal epithelial cell transplant) particularly PK (penetrating keratoplasty) procedures and the vision at most recent follow-up (AS = anatomical success, FS = functional success).

### Quality of assessment of the composite grafts

**Figure 4:**
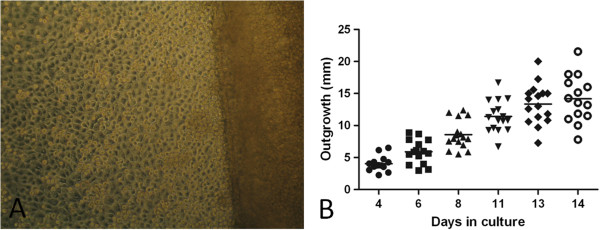
**Composite graft generation.** Phase contrast microscopy of a limbal biopsy and proliferating cells **(A)** (magnification × 200) and the outgrowth after the 14-day culture protocol **(B)** (n = 18).

**Figure 5:**
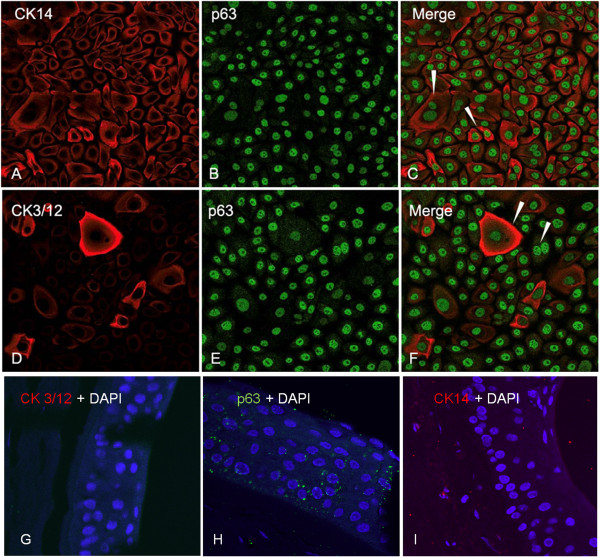
**Immunofluorescence microscopy of composite grafts.** Cells were stained for the presence of CK14 **(A)**, CK3/12 **(D)**, ∆Np63 **(B, E)**. **(C)** and **(F)** are composites of **(A, B)** and **(D, E)** respectively. Negative controls are shown in **(G)**, **(H)** and **(I)**.

In progenitor cell targeted CNT-20 culture medium, supplemented with 1% human blood type AB serum, biopsies showed an average outgrowth of 14.4 mm ± 3.7 mm by day 14 (Figure 4). The cells displayed a cobblestone epithelial phenotype when observed under phase contrast microscopy with a clear leading edge devoid of any cell debris. The grafts were assessed for the presence of a high percentage of progenitor cells using a panel of markers (Figure 5). The predominant phenotype (>50%) consisted of small cells with high nuclear-cytoplasmic ratio, showed positive expression for ABCG2, ∆Np63 and CK14 and negative expression for CK3/12 and desmoglein 3, all of which is consistent with a more immature progenitor phenotype. ABCG2 showed weak cytoplasmic and strong nuclear expression co-localizing with the transcription factor ∆Np63. VEGF-R1 and VEGF-R3 showed weak cytoplasmic staining (not all data is shown).

### Postoperative outcomes

**Figure 6:**
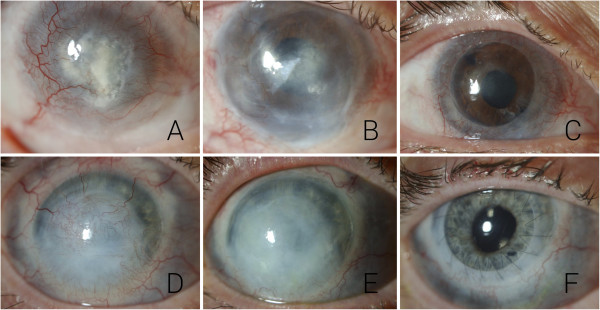
**Clinical images.** Representative images of patients that achieved anatomical success; pre op **(A + D)**, post LSCT **(B + E)** and post PK **(C + F)**.

**Figure 7:**
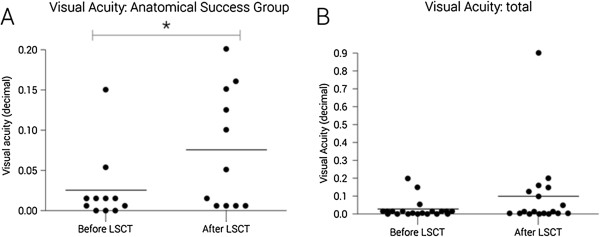
**Visual acuity.** Post-operative visual acuity outcomes in patients that achieved anatomical success **(A)** and for the total cohort **(B)** (* = p < 0.05).

**Figure 8:**
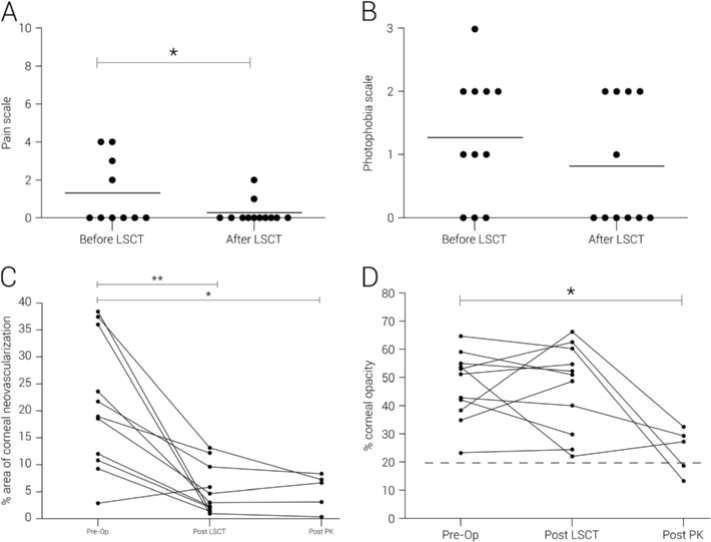
**Additional post-operative outcomes.** Post-operative outcomes in the anatomically successful groups: pain **(A)**, photophobia **(B)**, vascularization **(C)** and opacity **(D)**, (* = p < 0.05, **p < 0.01).

The transplant recipients were followed up for a mean of 22 months (range 4–43 months). Overall, 12 of the 18 recipients were graded anatomically successful (Table 1), resulting in a persistent continuous epithelial surface in 67% (95% CI:0.45 – 0.88). Representative images are displayed (Figure 6). Two of the 3 allogenic transplant recipients were graded as successful and 10 of the 15 autologous cases were deemed anatomically successful. A higher success rate was seen in women (p = 0.038) and a lower rate of success was seen in chemical burns (p = 0.013). Eight of the 12 patients were determined to be functional successes. Over the entire cohort we did not see a significant reduction in either pain or photophobia in the patient group post stem cell grafting. When we assessed the subgroup that achieved an anatomical success, there was a significant but modest improvement in visual acuity (p = 0.0289) with the exclusion of one outlier (Figure 7A). The outlying patient had a partial deficiency (Figure 7B) and the significant visual improvement was more attributed to the minimal involvement of the central cornea when compared with all of the other cases. When the group was assessed as a whole there was no significant increase in visual acuity (P = 0.065) (Figure 7B). In the success subgroup, there was a significant improvement in pain (p = 0.040) (Figure 8A) though there was no significant difference in photophobia (p = 0.029) (Figure 8B). Analysis of the ocular surface photographs showed that there was a significant reduction (p = 0.007) in the percentage area of corneal neovascularization in the successful subgroup (Figure 8C), which persisted in patients who underwent subsequent PK (p = 0.01). There was no significant influence on opacification after LSCT (p = 0.89) but there was a significant improvement seen after PK (p = 0.029) (Figure 8D). Seven patients in total received PKs, six to restore vision and one as a treatment for corneal perforation. Of these seven, the emergency graft was rejected and one of the six grafts to improve visual acuity was rejected and opacified (follow up range 7 – 36 months).

#### *Anatomical failures*

In the six cases of surgical failure, the graft failed to adhere and integrate with the hosts for a number of reasons. The first case (total limbal stem cell deficiency) showed primary failure of epithelialization and had reconjunctivalization of the cornea. Case number four had a period of initial integration followed by conjunctival overgrowth and had coexisting glaucoma. The fifth case had a stable enough surface to implant a PK but failed shortly thereafter. Patient 8 had recurrent epithelial defects and despite lubricants and a contact lens the defect did not close. The patient was noncompliant with clinic appointments thereafter. Patient number 10 showed conjunctival overgrowth but was incarcerated after 19 months of follow up. Since regular clinic attendances were not possible the patient was lost to further treatment. Patient 15 developed a perforation at the graft-host junction of a prior PK three days after LSCT. This was treated with an emergency PK, but in the absence of limbal stem cells, the graft failed.

## Discussion

In this study, we reported the results of a cultivated limbal stem cell protocol in a prospective phase I/II non-randomized clinical trial in 18 consecutive patients. The earliest clinical trials reported a culture protocol that included the use animal derived (xenogenic) products. Subsequent clinical trials have improved the patient safety profile of the technique by developing a xeno-free protocol. There is still variability in patient selection, culture techniques, donor materials and surgical technique across the reported studies and a widely accepted optimized protocol has not yet been developed. The presence of animal products such as mouse fibroblast feeder layers, FBS and cholera toxins carry potential health risks [22]. All of the bioengineered tissues produced using murine feeder cells are classified as xenografts by the US Food and Drug Administration (FDA) [23] and may incorporate nonhuman proteins such as sialic acids [24]. Animal products such as FBS have multiple constituents that vary in composition quality but may also be contaminated by endotoxins, haemoglobin, viruses, bacteria and prions.

In this clinical trial we aimed to optimize our approach by developing a standardized HAM preparation under xeno-free and stabilized culture conditions to generate the compostie grafts. We report that culturing LECs with serum–free CNT-20 on HAMs was unsuccessful, indicating that serum support is essential. We therefore substituted human serum for FBS, which has previously been shown to be successful in cultivating limbal stem cell grafts on HAM [6,7,13,14]. Clinical grade 1% human AB serum was an effective replacement for the bovine serum. Lekhanont et al. have also described a successful xeno-free technique of culturing corneal epithelial cells [25]. The Epilife medium used in this study, unlike CNT-20, is not specifically targeted towards proliferation of corneal progenitors. The CNT-20 protocol described here shows a higher proportion of progenitor cells with large nuclear to cytoplasmic ratios, and higher expression of ∆Np63+ and ABCG2+ than reported in the Epilife protocol. However these data are largely qualitative and further studies using quantitative analyses are warranted. We also reported a >8 mm diameter LEC outgrowth from 100% of biopsies placed in culture on standardized HAMs by day 14, compared with 70% outgrowth with Epilife [25].

The cell substrates are also known to play an important role in maintaining the contact microenvironment necessary for regulating cell behavior *in vitro*[26]. As such, we standardized our HAM to provide fixed, smooth membranes [15]. Amniotic membrane stabilization techniques have been described [27] but we customized the technique with a view to simplifying both the culture and surgical implantation protocols. Once the membrane is locked and the limbal explant seeded there is no need to touch or move the membrane. During surgery the composite graft can be sited and cut free with minimal manipulation reducing mechanical stress on the cultured cells and folds that predispose to primary failure of epithelialization [13]. In our centre only the first patient of the cohort experienced primary failure which may have been due to the learning curve associated with the technique. The five other failures occurred after an initial period of integration. Suture techniques may influence graft survival and promote vasculariszation [28-30]. In this protocol we reduce the influence of sutures by securing the composite graft with tissue fibrin glue and a temporary HAM patch. The patch, providing a reservoir of TGF-beta [19] is sutured under the free edge of the conjunctiva rather than to the cornea and any conjunctival overgrowth is discarded along with the patch at week one.

Anatomical success consistent with restoration of the corneal epithelium was seen in 67% of participants. While eighteen patients may represent a small population, the success rate is similar to that previously described [31]. Higher rates of rejection have been reported with allogenic transplantation [8] though this was not seen replicated in this study. Validation over a larger group would be required to substantiate this. Patients with chemical burns featured strongly in the failed graft cohort which is contrary to what has been reported in the literature. It is known that chronically inflamed eyes are at higher risk of graft failure [27], which may be the case in this cohort. Currently no standardized methods exist for detecting chronic or subclinical inflammation and assessment is usually made at the slit lamp on basis of vessel dilation. Further research into developing tools to detect inflammatory cytokines in tear samples may aid in determining inflammatory status of the eye prior to transplantation thereby improving overall outcome. Three cases of partial limbal stem cell deficiency were also included in the study as all three were persistent despite repeated surgical debridement and reconstruction. All three cases were found to achieve both anatomical and functional improvements, although including the partial cases increases the heterogeneity within the group. One-year post successful transplant, patients were offered a penetrating keratoplasty, which was performed in six cases for the purposes of visual improvement. In addition to the improvement in visual acuity, there was a significant decrease in corneal neovascularization seen. Impression cytology was only performed preoperatively to aid in diagnosis. Since cell pick-up was low and the procedure risked disrupting the transplanted epithelium, the test was not performed post operatively.

Objective assessments of corneal opacification have been developed [32-34] but these cannot be applied to LSCD cases as the backscatter is too high and renders the results unusable. The technique of opacity assessment here is based on analysis of slit lamp photographs, while not optimal, is a quantitative value of corneal haze that can be applied to these patients. At the time of surgery, the composite grafts contained both the cultured cells and the segment of the original limbal biopsy. This was based on the assumption that including the original biopsy in the graft would provide an additional benefit of the limbal niche. There was a concern however that the addition of this tissue could increase opacification. This was not observed and there was no significant effect of the graft procedure on central corneal opacity. Visual acuity improved after successful graft integration so this may indicate a reduction in opacity that is too subtle for this measurement technique to detect. Opacification did significantly improve after a PK procedure but the differences in clarity were great and relatively simple for the technique to detect. We conclude that this method is insufficiently sensitive to detect smaller changes in opacity and further work is required to develop a practical means of objectively determining corneal haze in this cohort.

In long-term limbal stem cell insufficiency, the chronic inflammatory process results in scarring that penetrates deep into the cornea. The replacement of the epithelial surface therefore has only a modest effect on vision. Limbal stem cell grafting therapy in these patients should not be considered sight-restorative. The aim is to provide a more normal epithelial surface and a safer platform on which to perform a PK that would improve vision.

## Conclusion

This study reports the results of a clinical trial with focus on building on the work of its predecessors. From biopsy to implantation we aimed to optimize the technique to remove the xenogenic components in culture, promote an undifferentiated morphology with CNT20 medium, standardize and stabilize the amniotic membranes with rings, simplify surgery and reduce any manipulation of the composite graft. We report the objective (visual acuity, slit lamp examination of the epithelium) and subjective outcomes (photophobia, pain) in our cohort and augment this with an objective assessment of area of neovascularization and opacification. We also provide a description outlining the features of the clinical failures. As the technique of cultured limbal stem cell grafting matures and is performed more widely, further optimization studies will be required in the future to achieve the perfect protocol for the best results in these complex patients.

## Competing interests

The authors have no competing interests to disclose.

## Author contributions

ZN: Conception and design, collection and assembly of data, data analysis and interpretation, manuscript writing. PT: Administrative support, provision of study material, collection and assembly of data. NDS: Manuscript preparation and writing. LI: Provision of study patients, administrative support. RJ: Collection and assembly of data. KC: Provision of study patients, administrative support. TJP: Data analysis and interpretation. BZ: Final approval of manuscript. TMJ: Conception and design, provision of study patients, final approval of manuscript. All authors read and approved the final manuscript.
